# A Novel Clinical-Radiomics Model Pre-operatively Predicted the Stone-Free Rate of Flexible Ureteroscopy Strategy in Kidney Stone Patients

**DOI:** 10.3389/fmed.2020.576925

**Published:** 2020-10-15

**Authors:** Yang Xun, Mingzhen Chen, Ping Liang, Pratik Tripathi, Huchuan Deng, Ziling Zhou, Qingguo Xie, Cong Li, Shaogang Wang, Zhen Li, Daoyu Hu, Ihab Kamel

**Affiliations:** ^1^Department of Urology, Tongji Hospital, Tongji Medical College, Huazhong University of Science and Technology, Wuhan, China; ^2^Department of Radiology, Tongji Hospital, Tongji Medical College, Huazhong University of Science and Technology, Wuhan, China; ^3^College of Life Science and Technology, Huazhong University of Science and Technology, Wuhan, China; ^4^Russell H. Morgan Department of Radiology and Radiological Science, the Johns Hopkins Medical Institutions, Baltimore, MD, United States

**Keywords:** clinical-radiomics model, flexible ureteroscopy, kidney stone, computed tomography, lithotripsy

## Abstract

**Purpose:** The purpose of the study is to develop and validate a novel clinical–radiomics nomogram model for pre-operatively predicting the stone-free rate of flexible ureteroscopy (fURS) in kidney stone patients.

**Patients and Methods:** Altogether, 2,129 fURS cases with kidney stones were retrospectively analyzed, and 264 patients with a solitary kidney stone were included in a further study. For lower calyx calculi, a radiomics model was generated in a primary cohort of 99 patients who underwent non-contrast-enhanced computed tomography (NCCT). Radiomics feature selection and signature building were conducted by using the least absolute shrinkage and selection operator (LASSO) method. Multivariate logistic regression analysis was employed to build a model incorporating radiomics and potential clinical factors. Model performance was evaluated by its discrimination, calibration, and clinical utility. The model was internally validated in 43 patients.

**Results:** The overall success rate of fURS was 72%, while the stone-free rate (SFR) for lower calyx calculi and non-lower calyx calculi was 56.3 and 90.16%, respectively. On multivariate logistic regression analysis of the primary cohort, independent predictors for SFR were radiomics signature, stone volume, operator experience, and hydronephrosis level, which were all selected into the nomogram. The area under the curve (AUC) of clinical–radiomics model was 0.949 and 0.947 in the primary and validation cohorts, respectively. Moreover, the calibration curve showed a satisfactory predictive accuracy, and the decision curve analysis indicated that the nomogram has superior clinical application value.

**Conclusion:** In this novel clinical–radiomics model, the radiomics scores, stone volume, hydronephrosis level, and operator experience were crucial for the flexible ureteroscopy strategy.

## Introduction

As a common urological disease, the prevalence rates for kidney stones vary from 1 to 20%. Especially in western developed countries such as the USA, kidney stone prevalence is notably high (>10%) (1). Related treatment costs are estimated to be several billion dollars per year in such countries (2). With the continuous development of the flexible ureteroscope and auxiliary equipment, flexible ureteroscopy (fURS) is considered to be one of the first-line treatments for the active removal of renal stones smaller than 2 cm (3). Furthermore, recent studies have shown fURS to be less dangerous for severe complications than percutaneous nephrolithotomy (PCNL) and also has low possibility for retreatment compared with shock wave lithotripsy (SWL) (4, 5).

However, the complete stone-free rate (SFR) for fURS is relatively low compared with PCNL (6). Numerous factors such as stone characteristics, stone location, renal anatomy, and hydronephrosis level may affect the success rate of fURS. Among these factors, the characteristics of the stone are very crucial. Several articles documented that the composition or Hounsfield units (HU) of the stone have a great impact on the efficacy of fURS (7, 8). However, HU based on computed tomography (CT) only represents the average value of the stone and thus cannot reflect the intracalculi structure, which is eminently related to the SFR. In addition, our previous research discovered that CT texture analysis (CTTA) of urinary tract stones may better predict the SFR on ESWL patients (9). Apparently, the limitations of this previous study included solitary enrollment of simple first-order parameters, and failure to combine clinical factors and lack multivariate analysis.

Recent advances in computer-assisted imaging techniques have enabled the high-throughput extraction of quantitative features from digital medical images. Actually, this new methodology, named radiomics, has been proven to be capable of influencing and altering the diagnosis and treatment strategies in the field of tumors (10, 11). Moreover, several investigations showed that predictive model based on radiomics or machine learning can better predict the post-operative outcome of certain surgical treatments (PCNL or SWL) (12, 13). It is important to develop a novel predictive model for fURS that combines radiomics features and clinical indicators, particularly for the lower renal stones.

Accordingly, the objective of this study was to develop and validate a novel clinical–radiomics nomogram model for pre-operative assessment and prediction SFR of fURS.

## Materials and Methods

### Patients

Ethical approval was obtained for this retrospective analysis, and the requirement for informed consent was waived. In the present study, we retrospectively enrolled 2,129 fURS for renal stone removal performed between December 2014 and March 2019 (Supplementary Figure 1 shows flowchart of patient selection). In total, 264 patients with solitary kidney stone met the inclusion criterion, including 122 cases of non-lower calyx calculi and 142 cases of lower calyx calculi. Lower calyx calculi treated with fURS were included in further research and randomly divided into two independent cohorts: primary cohort and validation cohort with a ratio of 7:3 based on the 10-fold cross-validation principle.

Meanwhile, patients' pertinent clinical data and stone characteristics were extracted pre-operatively, including age, sex, hydronephrosis level, the burden of calculi, pre-operative catheterization, the experience of the surgeon, etc. The follow-up procedure was conducted on the basis of the results of the CT scan or X-ray of kidney–ureter–bladder (KUB) review 3 months after operation. The standard of stone-free status was defined as free from stones or residual stone fragments <2 mm (14).

### Surgical Techniques

Rigid ureteroscopy was routinely used for ureteral dilation before fURS. Thereafter, a 0.035-mm straight guidewire was placed through the ureteric orifice to the renal pelvis under direct rigid ureteroscope vision. Then, we placed a 14-F ureteric access sheath (Cook Medical, Bloomington, IN, USA) by a straight guidewire. A 7.5-F flexible ureteroscope (Flex-X2, Karl Storz, Germany) was passed through the ureteric access sheath to access the stone. Once the location of stones was confirmed, the Ho:YAG laser was used to fragment stones. After lithotripsy, 6-F double-J stent was routinely left in all cases for 2–4 weeks.

### CT Image Acquisition, Region of Interest Segmentation, and Radiomics Feature Extraction

All included patients underwent NCCT using a 64-slice MDCT scanner (Discovery CT750 HD, GE Healthcare, USA). The related CT imaging acquisition parameters are as follows: tube voltage, 100–120 kV; automatic tube current, 200–350 mA; rotation time, 0.5 s; scan slice thickness, 5 mm; and reconstruction thickness, 1.25 mm.

Stone regions of interest (ROIs) were manually segmented on each transverse slice CT images, in the format of DICOM, using an open-source software 3D Slicer (version 4.9.0; www.slicer.org). Then, the following features were documented: (a) stone size, defined as maximum diameter on images; (b) stone volume, the ROIs would be fused and become the volume of interest (VOI); (c) stone location, subclassified as lower and non-lower calyx calculi; and (d) the degree of hydronephrosis, defined as severe and non-severe.

Radiomics feature extraction was performed using in-house texture analysis software with algorithms implemented in Matlab 2015a (MathWorks, Natick, Mass). In our research, a total of 604 radiomics features, including first-order statistics, shape- and size-based features, textural features, and wavelet features, were generated from each original CT image. Specific details of feature algorithms are shown in Supplement 1 in Supplementary Material.

### Radiomics Feature Selection and Signature Construction

Dimension reduction and signature building process were arranged by LASSO logistic regression algorithm (15). With penalty parameter tuning conducted by 10-fold cross-validation, LASSO was performed to select robust and non-redundant features from the primary cohort. A radiomics signature was created by a linear combination of selected features weighted by their respective coefficients, and the relevant radiomics score (Rad-score) was calculated for each patient.

### Development, Performance, and Validation of a Clinical–Radiomics Nomogram Model

A model that incorporated the radiomics signature and clinical factors for predicting stone-free (SF) status was built based on multivariate logistic regression analysis in the primary set. We initially excluded some variables from the multivariate model for multicolinearity using variance inflation factor (VIF).

To provide a visual tool for clinical decision-making, a clinical–radiomics nomogram was then generated based on multivariate logistic regression analysis of corresponding pre-operative factors. Model performance was typically measured in terms of discrimination and calibration. The area under the curve (AUC), calculated by receiver operating characteristic (ROC) curve, was used to quantify the discrimination performance of established models (16). Calibration curves were portrayed to evaluate the predictive accuracy of the clinical–radiomics nomogram, followed by the Hosmer–Lemeshow goodness-of-fit test (a significant test statistic means that the model does not calibrate perfectly) (17).

To evaluate its predictive accuracy, the performance of nomogram was tested in the validation cohort. The logistic regression formula formed in the primary cohort was applied to all patients of the validation cohort, with total points for each patient calculated. Finally, the ROC and calibration plot were generated based on the regression analysis.

### Clinical Utility of the Clinical–Radiomics Nomogram Model

Finally, to determine the clinical value of the radiomics model that incorporates clinical consequences, the decision curve analysis (DCA) was conducted to demonstrate a quantification of the net benefits at different threshold probabilities (18).

### Statistical Analysis

Statistical analysis was conducted with Statistical Package for Social Sciences (SPSS) software version 24.0 and R (version 3.4.4) with R packages listed in Supplement 2 in Supplementary Material. The normality of all continuous variables was evaluated by the Kolmogorov–Smirnov test. Univariate analysis (chi-square test for categorical variables and *t*-test or rank sum test for continuous variables) and multivariate statistical analysis (logistic regression) were performed to identify significant independent predictors. A two-sided *p* < 0.05 was described as significant.

## Results

### Clinical Characteristics

Baseline characteristics of all patients are shown in Table 1. A total of 264 patients, 160 (60.6%) men and 104 (39.4%) women, were enrolled in this study. The overall rate of SF was about 72%. Significant difference was revealed between the SF and non-SF groups in the following indicators: stone location (*p* < 0.001), hydronephrosis (*p* < 0.001), operator experience (*p* < 0.001), and stone volume (*p* < 0.001).

**Table 1:** Comparison of single kidney stone patient and stone characteristics according to SF at 3 months after fURS.

**Variable**	**SF**	**Non-SF**	***p***
Number of patients, *n*	190	74	
Age, mean ± SD, years	49.26 ± 12.00	49.12 ± 11.45	0.933^a^
**Gender**, ***n*** **(%)**			0.244
Male	111 (69.4%)	49 (30.6%)	
Female	79 (76.0%)	25 (24.0%)	
BMI, mean ± SD, kg m^−2^	23.71 ± 3.35	23.53 ± 3.49	0.778^a^
**Pre-operative stenting**, ***n*** **(%)**			0.312
No	168 (73.0%)	62 (27.0%)	
Yes	22 (64.7%)	12 (35.3%)	
**Pre-operative ESWL**, ***n*** **(%)**			0.884
No	163 (71.8%)	64 (28.2%)	
Yes	27 (73.0%)	10 (27.0%)	
**History of stone surgery**, ***n*** **(%)**			0.097^b^
No	160 (74.1%)	56 (25.9%)	
fURS	15 (71.4%)	6 (28.6%)	
PCNL	7 (43.8%)	9 (56.3%)	
Ureterolithotomy	4 (80%)	1 (20%)	
Pyelolithotomy	4 (66.7%)	2 (33.3%)	
**Hypertension**, ***n*** **(%)**			0.677
No	147 (71.4%)	59 (28.6%)	
Yes	43 (74.1%)	15 (25.9%)	
**Diabetes**, ***n*** **(%)**			0.795
No	179 (71.6%)	71 (28.4%)	
Yes	11 (78.6%)	3 (21.4%)	
**Stone laterality**, ***n*** **(%)**			0.089
Left	96 (67.6%)	46 (32.4%)	
Right	94 (77.0%)	28 (23.0%)	
**Stone location**, ***n*** **(%)**			<0.001
Lower calyx	80 (56.3%)	62 (43.7%)	
Non-lower calyx	110 (90.2%)	12 (9.8%)	
**Hydronephrosis**			<0.001
No/mild	168 (87.5%)	24 (12.5%)	
Severe	22 (30.6%)	50 (69.4%)	
**Experience of operator**, ***n*** **(%)**			<0.001
fURS ≥ 100	115 (87.8%)	16 (12.2%)	
fURS < 100	75 (56.4%)	58 (43.6%)	
**Stone diameter (cm)**, ***n*** **(%)**			0.067
≤ 1	106 (76.8%)	32 (23.2%)	
>1	84 (66.7%)	42 (33.3%)	
**Stone volume (cm**^**3**^**)**, ***n*** **(%)**			<0.001
≤ 1	134 (79.8%)	34 (20.2%)	
>1	56 (58.3%)	40 (41.7%)	

*SD, standard deviation; fURS, flexible ureteroscope; SF, stone free*.

a *t-test*.

b *Rank sum test*.

*Others are chi-square test*.

However, in the subgroup analysis of stone location, the SFR for lower calyx calculi and non-lower calyx calculi was 56.3% (80/142) and 90.16% (110/122), respectively (Table 2). In the lower calyx group, stone diameter, hydronephrosis, experience of operator, and stone volume (all *p* < 0.001) were found to be the significant factors effecting fURS results, while SF status was significantly associated with hydronephrosis (*p* = 0.008) and experience of operator (*p* = 0.003) in the non-lower calyx group.

**Table 2:** Influencing factors on the success rate after fURS between lower calyx stone and non-lower calyx stone patients.

	**Lower calyx**			**Non-lower calyx**	
	**SF**	**Non-SF**	***p***	**SF**	**Non-SF**	***p***
Number of patients, *n*	80	62		110	12	
Age, mean ± SD, years	50.15 ± 11.42	48.85 ± 12.01	0.513^a^	48.61 ± 12.42	50.50 ± 8.22	0.608^a^
**Gender**, ***n*** **(%)**			0.682			0.004
Male	45 (54.9%)	37 (45.1%)		66 (84.6%)	12 (15.4%)	
Female	35 (58.3%)	25 (41.7%)		44 (100%)	0 (0%)	
BMI, mean ± SD, kg m^−2^	23.97 ± 3.38	23.34 ± 3.49	0.367^a^	23.10 ± 2.99	24.84 ± 1.06	0.418^a^
**Stone laterality**, ***n*** **(%)**			0.151			0.952
Left	42 (51.2%)	40 (48.8%)		54 (90.0%)	6 (10.0%)	
Right	38 (63.3%)	22 (36.7%)		56 (90.3%)	6 (9.7%)	
**Hydronephrosis**			<0.001			0.008
No/mild	66 (79.5%)	17 (20.5%)		75 (96.2%)	3 (3.8%)	
Severe	14 (23.7%)	45 (76.3%)		35 (79.5%)	9 (20.5%)	
**Experience of operator**, ***n*** **(%)**			<0.001			0.003
fURS ≥ 100	57 (79.2%)	15 (20.8%)		58 (98.3%)	1 (1.7%)	
fURS < 100	23 (32.9%)	47 (67.1%)		52 (82.5%)	11 (17.5%)	
**Stone diameter (cm)**, ***n*** **(%)**			<0.001			0.796
≤ 1	60 (68.2%)	28 (31.8%)		46 (92.0%)	4 (8.0%)	
>1	20 (37.0%)	34 (63.0%)		64 (88.9%)	8 (11.1%)	
**Stone volume (cm**^**3**^**)**, ***n*** **(%)**			<0.001			0.890
≤ 1	68 (65.4%)	36 (34.6%)		66 (89.2%)	8 (10.8%)	
>1	12 (31.6%)	26 (68.4%)		44 (91.7%)	4 (8.3%)	

*SD, standard deviation; fURS, flexible ureteroscope; SF, stone free*.

a *t-test*.

*Others are chi-square test*.

Subsequently, we further conducted studies on the lower calyx cases. Ninety-nine patients were included in primary groups to establish a predictive model, and 43 patients were enrolled in the validation groups to verify the accuracy and reliability of the generated model. Both two groups were further divided into SF and non-SF cases separately in accordance with the results of follow-up of each patient. Univariate analysis revealed the possible association in five factors (hydronephrosis level, operator experience, stone diameter, stone volume, and radiomics score) and the SFR, as presented in Table 3.

**Table 3:** Characteristics of lower calyx stone patients in the primary and validation cohorts.

	**Primary cohort**			**Validation cohort**	
	**SF**	**Non-SF**	***p***	**SF**	**Non-SF**	***p***
Number of patients, *n*	56	43		24	19	
Age, mean ± SD, years	50.34 ± 10.82	48.40 ± 12.31	0.406^a^	49.71 ± 12.94	49.89 ± 11.55	0.961^a^
**Gender**, ***n*** **(%)**			0.350			0.553
Male	34 (53.1%)	30 (46.9%)		11 (61.1%)	7 (38.9%)	
Female	22 (62.9%)	13 (37.1%)		13 (52.0%)	12 (48.0%)	
BMI, mean ± SD, kg m^−2^	24.64 ± 3.75	23.15 ± 3.76	0.136^a^	23.71 ± 3.24	24.12 ± 3.20	0.754^a^
**Stone laterality**, ***n*** **(%)**			0.056			0.759
Left	27 (48.2%)	29 (51.8%)		15 (57.7%)	11 (42.3%)	
Right	29 (67.4%)	14 (32.6%)		9 (52.9%)	8 (47.1%)	
**Hydronephrosis**			<0.001			<0.001
No/mild	46 (78.0%)	13 (22.0%)		20 (83.3%)	4 (16.7%)	
Severe	10 (25.0%)	30 (75.0%)		4 (21.1%)	15 (78.9%)	
**Experience of operator**, ***n*** **(%)**			<0.001			0.001
fURS ≥ 100	40 (78.4%)	11 (21.6%)		17 (81.0%)	4 (19.0%)	
fURS < 100	16 (33.3%)	32 (66.7%)		7 (31.8%)	15 (68.2%)	
**Stone diameter (cm)**, ***n*** **(%)**			0.007			0.012
≤ 1	42 (66.7%)	21 (33.3%)		18 (72.0%)	7 (28.0%)	
>1	14 (38.9%)	22 (61.1%)		6 (33.3%)	12 (66.7%)	
**Stone volume (cm**^**3**^**)**, ***n*** **(%)**			<0.001			0.002
≤ 1	49 (71.0%)	20 (29.0%)		19 (76.0%)	6 (24.0%)	
>1	7 (23.3%)	23 (76.7%)		5 (27.8%)	13 (72.2%)	
Radiomics score, median (interquartile range)	1.348 (0.467–2.243)	−0.462 (−1.429 to 0.260)	<0.001^b^	0.557 (−0.175 to 2.009)	−0.895 (−1.482 to −0.119)	<0.001^b^

*SD, standard deviation; fURS, flexible ureteroscope; SF, stone free*.

a *t-test*.

b *rank sum test*.

*Others are chi-square test*.

### Feature Selection, Radiomics Signature Construction, and Validation

**Figure 1:**
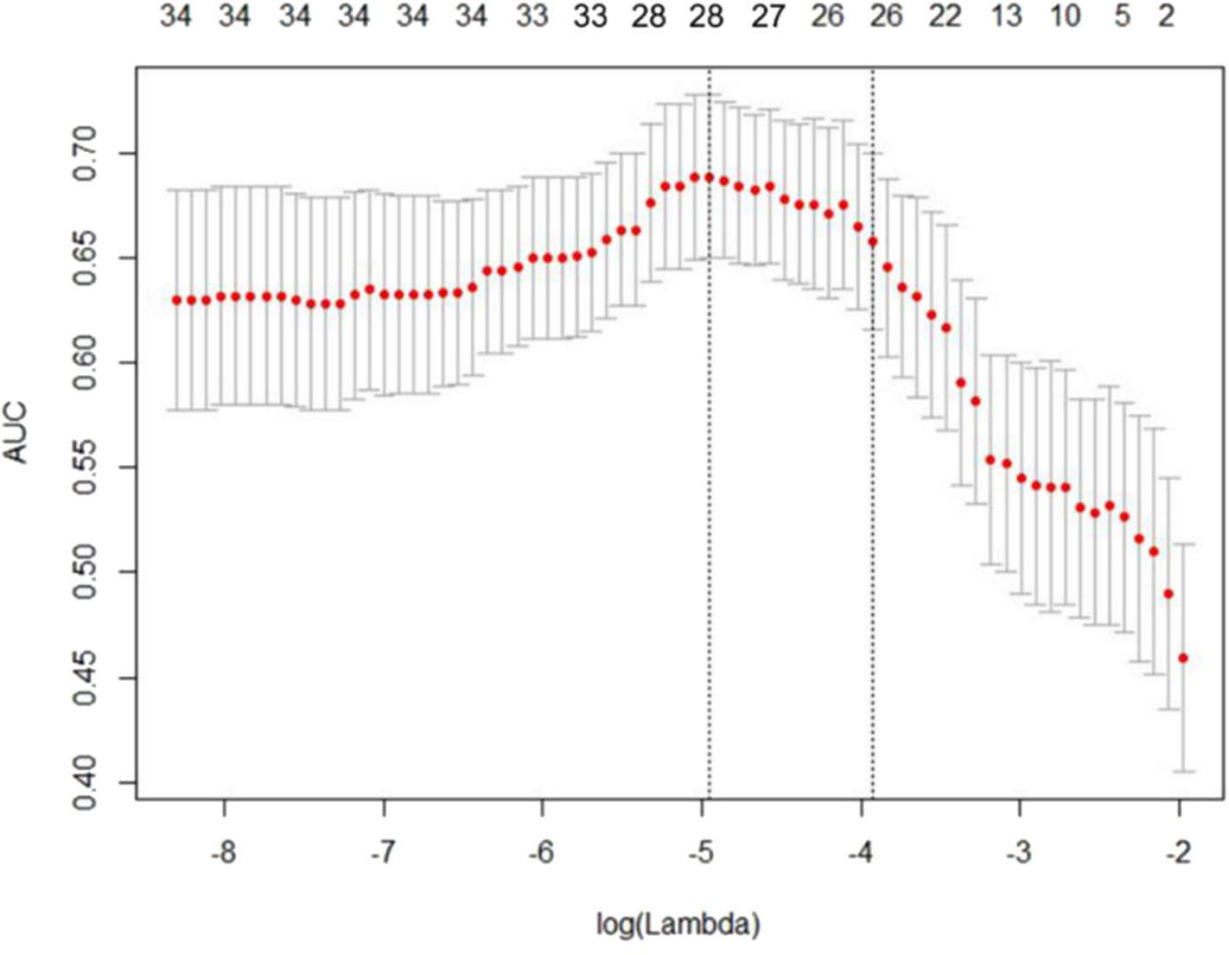
Least absolute shrinkage and selection operator (LASSO) regression analysis uses the minimum standard and a 10-fold cross-validation method. The coefficients of the model are compressed by introducing a penalty adjustment parameter (λ) so that the coefficients of the irrelevant variables tend to be zero, and then, the automatic screening of the variables is realized.

Using the 604 extracted radiomics features, LASSO analysis was performed, and 28 features with non-zero coefficients were screened based on the primary group (Figure 1).

A radiomics score calculation formula was then constructed by using corresponding coefficients of the chosen signature, presented in Supplement 3 in Supplementary Material. Distributions of the radiomics score and post-operative outcome for each patient in the primary and validation groups are shown in Supplementary Figure 2.

A significant difference in radiomics score was initially evidenced between SF and non-SF patients in the primary group (*p* < 0.001), and later confirmed in the validation group (*p* < 0.001), which can be noted in Table 3. The results show that the index based on radiomics analysis is significantly and positively correlated with post-operative SFR. Namely, patients with an SF outcome generally had higher Rad scores in the primary cohort.

### Development, Performance, and Diagnostic Validation of Prediction Models

**Figure 2:**
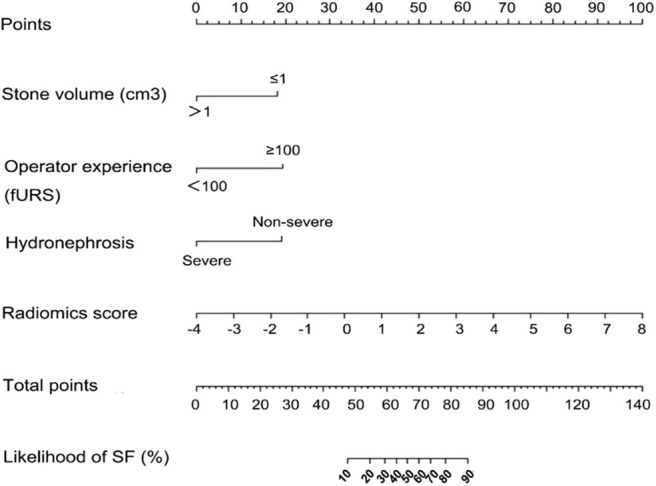
Established clinical–radiomics nomogram model. The clinical–radiomics nomogram was generated in the primary cohort, with the radiomics signature, stone volume, operator experience, and hydronephrosis level incorporated.

Results of multivariate regression analysis are shown in Table 4. The VIFs of three potential predictors ranged from 1.2 to 1.42, showing that there was no multicolinearity. A radiomics model that is composed of four different parameters (stone volume, operator experience, hydronephrosis level, and radiomics signature) was constructed and presented as a nomogram (Figure 2). Higher total point reflects corresponding case with higher probability for post-operative treatment success.

**Table 4:** Multivariate analysis of the influencing factors on the success rate after fURS in lower calyx patients.

**Variables**	**B**	**SE**	**OR**	**95% CI**	***p***
Male	−0.045	0.746	0.956	0.222–4.129	0.952
Left	−1.328	0.758	0.265	0.060–1.170	0.080
No/mild hydronephrosis	2.293	0.762	9.908	2.224–44.132	0.003
Stone volume ≤ 1 cm^3^	2.121	0.945	8.337	1.309–53.106	0.025
fURS ≥ 100	2.413	0.854	11.169	2.095–59.550	0.005
Radiomics score	0.986	0.333	2.679	1.395–5.146	0.003

*fURS, flexible ureteroscope*.

**Figure 3:**
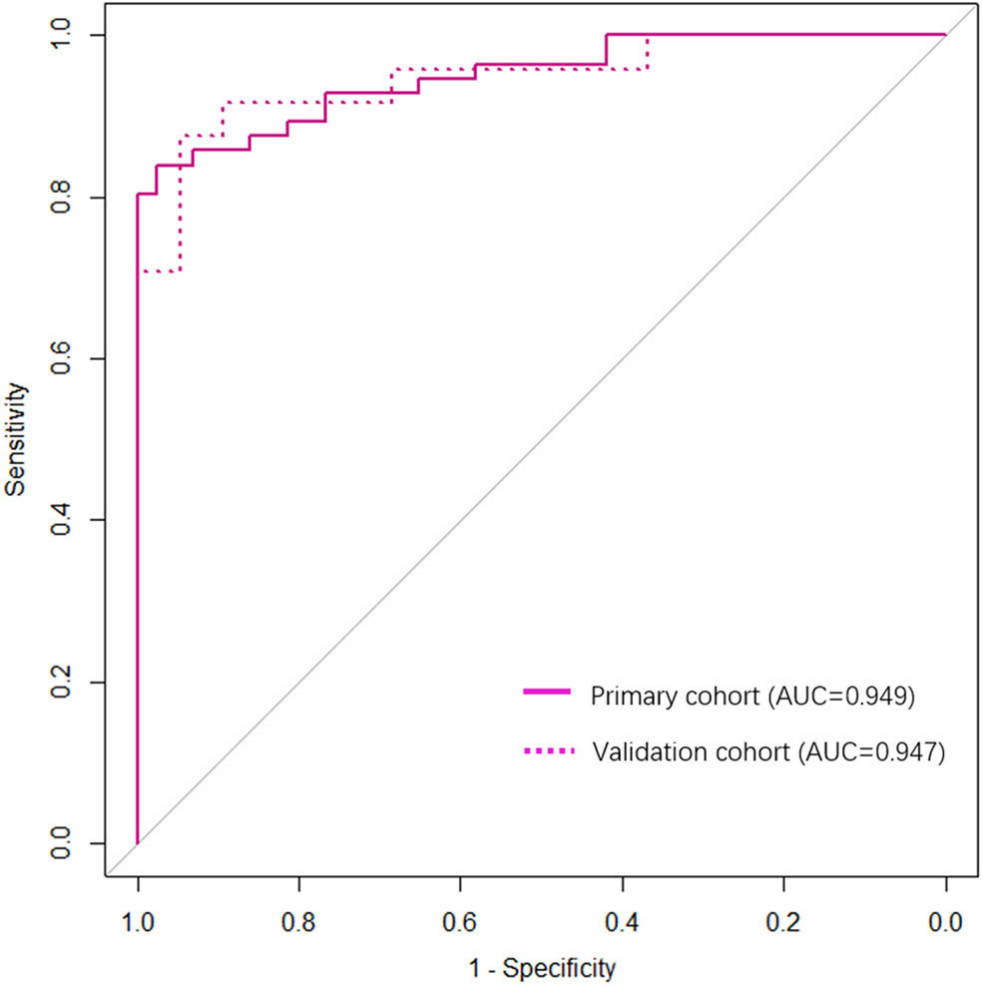
Receiver operating characteristic (ROC) based on clinical–radiomics nomogram.

**Figure 4:**
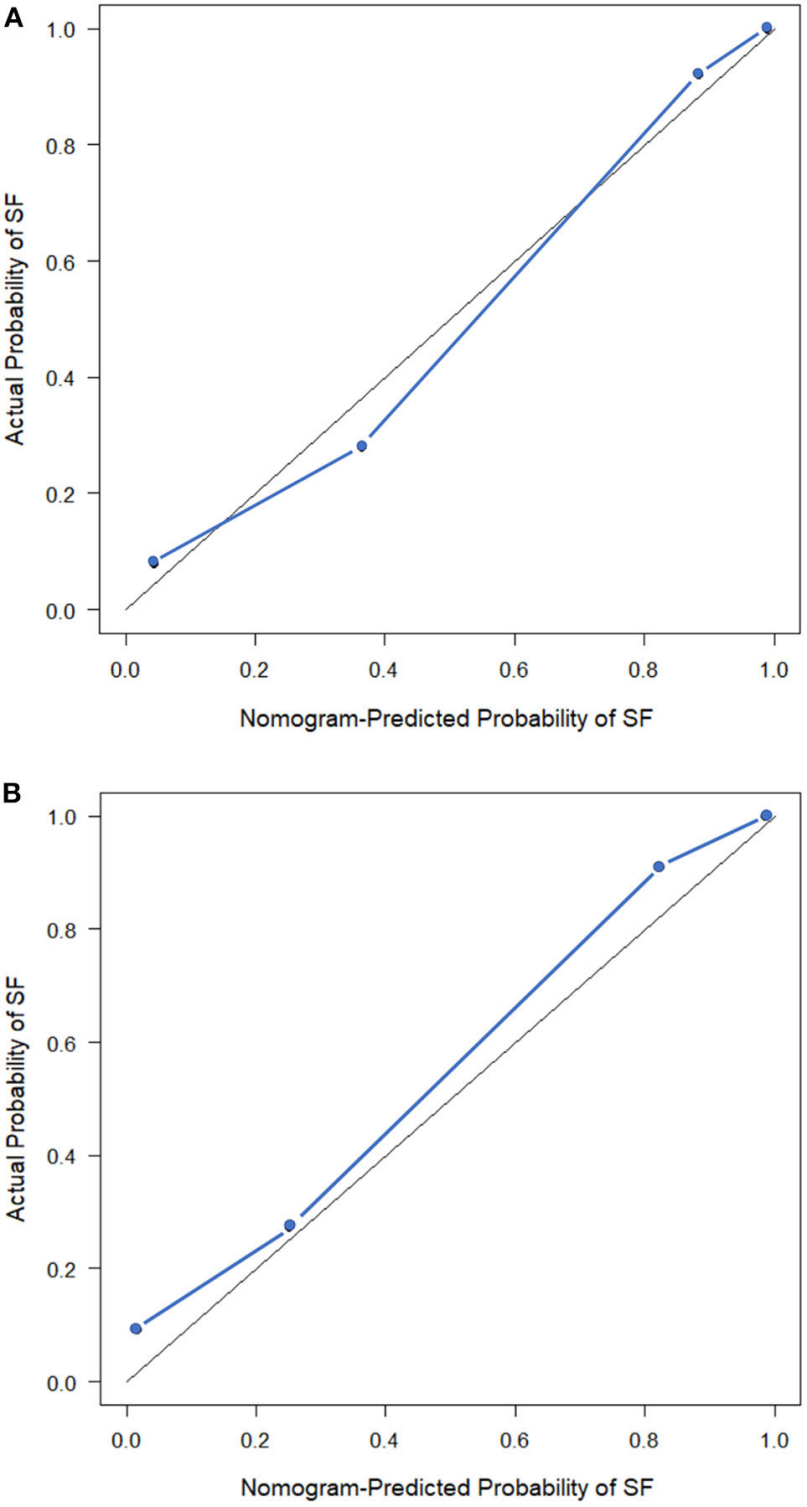
Calibration curves of the clinical–radiomics nomogram. **(A)** Calibration curve of the clinical–radiomics nomogram in the primary cohort. **(B)** Calibration curve of the clinical–radiomics nomogram in the validation cohort. Calibration curves describe the calibration of the model with respect to the agreement between nomogram predicted probability of stone free (SF) and actual SF rate. The y-axis depicts actual SF rate. The x-axis depicts the nomogram predicted probability of SF. The diagonal solid line depicts an excellent prediction by a supreme model. The blue dotted line represents the performance of the nomogram.

**Figure 5:**
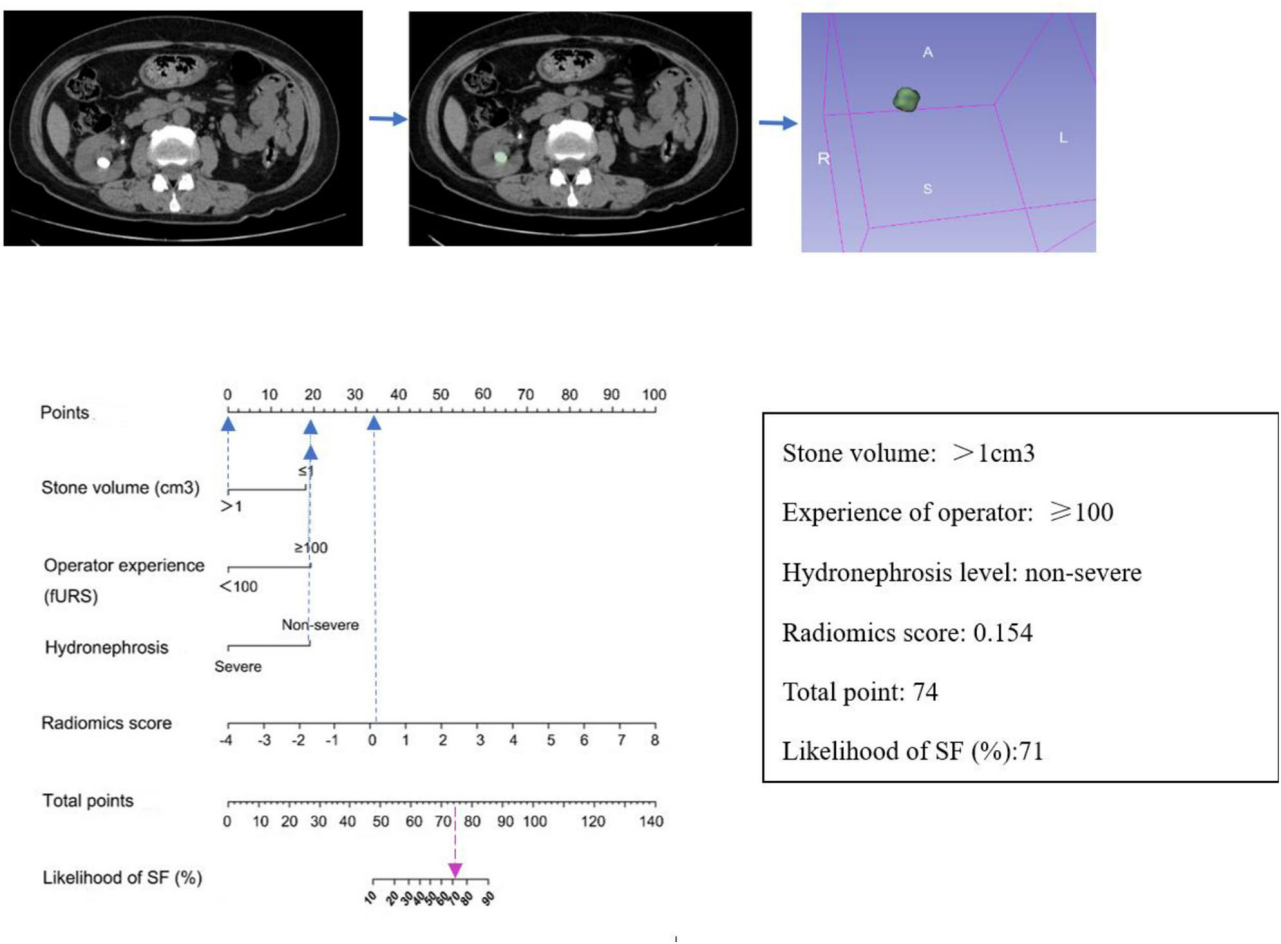
An example of how to use the clinical–radiomics nomogram to predict SF status in a 58-year-old female patient with SF outcome after flexible ureteroscopy (fURS). Locate the patient's Rad-score on the Rad-score axis. Draw a line straight upward to the points' axis to determine how many points the patient receives for his or her Rad-score. Conduct a similar process for other indicators. Sum the points calculated for each of the risk factors and track down the added sum on the total points axis. Draw a line straight down to find the patient's likelihood of SF.

The observed AUC value for nomogram predictions was 0.949 (95% CI, 0.910–0.989). The established nomogram was then verified in a validation cohort, with AUC of 0.947 (95% CI, 0.883–1) supported the increased predictive efficacy (Figure 3). The calibration curve and the Hosmer–Lemeshow test (*p* = 0.344) demonstrated favorable calibration of the nomogram in the primary group (Figure 4A). In the validation cohort, the calibration curve showed that there was a good agreement between nomogram predicted probability of SF and actual SF rate (Figure 4B). Meanwhile, the Hosmer–Lemeshow test yielded a non-significant statistic (*p* = 0.099), which suggested that there was no departure from perfect fit. Figure 5 showed a specific clinical case decision procedure utilizing the generated clinical–radiomics nomogram.

### Clinical Use

**Figure 6:**
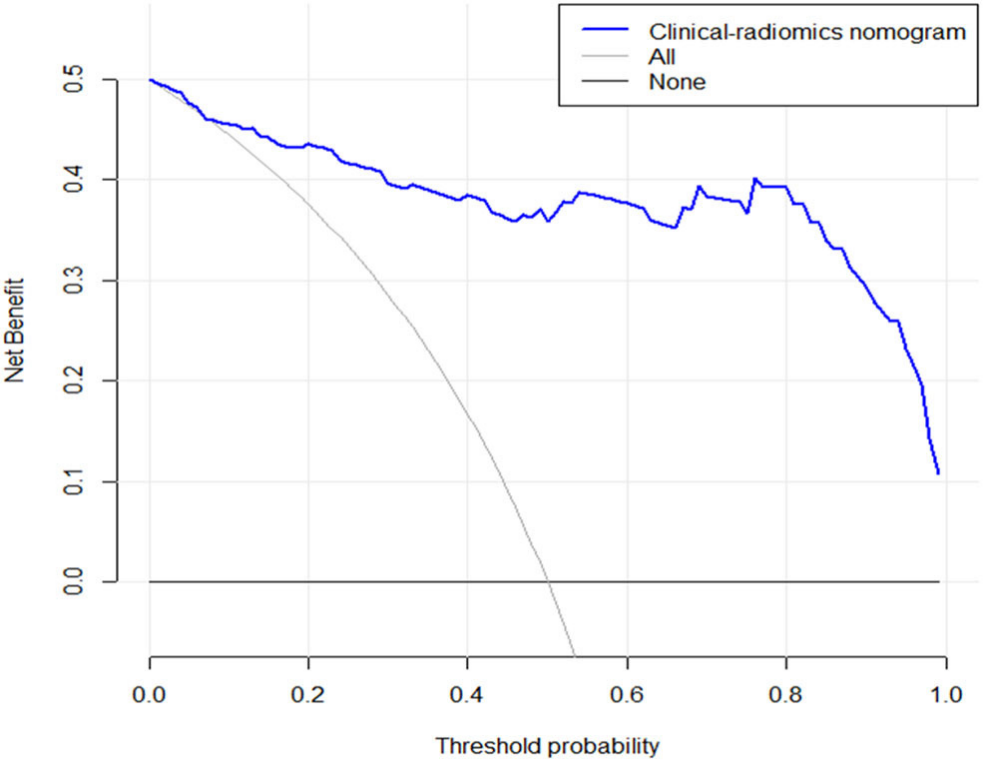
The y-axis measures the net benefit. The blue line represents the clinical–radiomics nomogram. The gray line represents the assumption that all patients have SF outcome. The black line represents the presumption that no patients have SF status. The decision curve showed that if the threshold probability is above 0.1, then using an imaging colinearity chart to predict post-operative result is more beneficial for making clinical decisions. For example, if the personal threshold probability of a patient is 50% (i.e., the patient would opt for treatment if his probability of SF was 50%); then, the net benefit is 0.36 when using the clinical–radiomics nomogram to make the decision of whether to undergo treatment, with added benefit than the treat-all scheme or the treat-none scheme).

The DCA for the clinical–radiomics nomogram is portrayed in Figure 6. The decision curve showed that if the threshold probability of a patient is above 0.1, using the predictive model to predict SFR provides a better net benefit than either the treat-all-patients scheme or the treat-none scheme.

## Discussion

In recent years, fURS has become a mainstream surgical treatment for upper urinary calculi with its advantages of non-invasiveness, safety, and having a short learning curve. The indications for fURS have gradually expanded, and some scholars have even tried to use it for the treatment of staghorn calculi. It is very likely to be an alternative to ESWL and PCNL and to become the preferred surgical procedure for upper urinary calculi (19–21). After the craze, some urologists began to reflect on whether this technique is so widely applicable. It is undeniable that some patients have unsatisfactory results after receiving fURS and even fail to touch the stones, causing patients to undergo ineffective surgery and need to be treated again, which brings huge safety risks and waste of medical resources (22). Therefore, it is particularly important to pre-operatively assess the effect of fURS surgery and accurately select patients who are suitable for fURS.

Stone characteristics have been considered as a significant factor determining the final efficacy because various stone characteristics can result in different times and extents of pulverization under the function of laser (7, 8, 23). Stones with longer pulverization times are more likely to reposition during the process, which may lead to an unsuccessful outcome. However, comprehensively identifying stone characteristics prior to surgery still remains an intractable challenge. On the one hand, traditional stone composition analysis can only depend on post-operative or intraoperative *vitro* testing, which is not feasible for pre-operative assessment. On the other hand, simple measurement of Hounsfield unit or density is unilateral, since the situation of intracalculi is often uneven and complicated. This explains why many studies failed to include this crucial factor into the evaluation scoring system (24, 25).

In this study, a radiomics signature consisting of 28 robust features was identified to be an independent factor for the SFR of fURS in patients with the lower calyx calculi. This multifeature-based radiomics signature also successfully stratified patients into successful and unsuccessful groups in the validation dataset. Our previous study on SWL also found that CTTA, a quantitative analysis method, may be useful in improving medical decision-making on ESWL patients (9). Similarly, several pieces of research showed that establishing a prediction model utilizing radiomics or machine learning may contribute to a better predictive efficacy for pre-operative estimation of PCNL or SWL outcomes (12, 13).

Obviously, the radiological features alone were not enough, and it has been considered that relying on a solitary strong risk indicator could fail to evaluate the comprehensive post-operative outcome of individual patients (26). Therefore, we generated a clinical–radiomics model, which is the combination of the radiomics signature and potential clinical indicators. The established clinical–radiomics nomogram demonstrated superior discrimination and calibration in both training and validation cohort, with an AUC of 0.949 and 0.947, respectively. Likewise, the decision curve analysis indicated that the clinical–radiomics nomogram was more beneficial than the treat-all scheme or the treat-none scheme across the majority of the scope of rational threshold probabilities.

Indeed, with respect to clinical predictors, there are some similarities between our study and Ito's original study (24). The two studies both agreed that the location, stone volume, hydronephrosis level, and surgeon's experience are significant predictors of post-operative SF status. Many articles have reported that the stone volume greatly affects the success rate of fURS (27), and our research also showed similar results. With the increase in stone burden, it would require a longer pulverization time, and the stone fragments are more likely to move. At the same time, it may increase the probability of intraoperative bleeding, which can lead to blurred vision, affecting normal surgical procedure and thus leading to stone residues after surgery. In addition, hydronephrosis causes enlargement of the renal pelvis and calyces, which makes breaking and basketing stones trickier, thus increasing the likelihood of stone residues after the procedure. Furthermore, Ito et al. reported on urologists with experience of >100 fURS that were associated with a satisfactory post-operative outcome. Meanwhile, Cho et al. reported that 56 cases were required for reaching a plateau in the learning curve (28). Another study indicated that surgeon experience affects the outcomes of fURS mainly in terms of safety (29). Similarly, in our department, we found that operators with experience of more than 100 procedures can achieve more skilled surgical techniques and deal with complications more efficiently. Being proficient in using ureteroscopy and its ancillary equipment, they can reduce some dangerous complications, such as intraoperative bleeding, or even prevent them from happening.

Unlike prior prognostic investigations that mostly analyzed all kinds of patients regardless of stone location, our current study focused exclusively on patients with the lower calyx calculi. Among various treatments for single lower calyceal stone, Bozzini et al. (30) reported that fURS and PCNL were more effective than SWL to obtain a better SFR and a lower auxiliary and retreatment rate. fURS, compared with PCNL, offers the best outcome in terms of procedure length, radiation exposure, and hospital stay. De et al. (31) suggested that PCNL provides overall significantly higher stone-free rates than fURS, at the expense of higher complication rates, blood loss, and a longer length of stay. Nevertheless, fURS can provide higher stone-free rates compared with minimally invasive percutaneous procedures. In our study, the overall rates of SF of fURS process was about 72%, which is consistent with other studies (ranging between 65 and 92%) (32). However, in the subgroup analysis based on location, the SF rate for the lower calyx calculi only reached 56.3%, which was obviously lower than the figure for the non-lower calyx calculi (90.16%). It is also verified in another study that the stone treatment in the lower pole is less effective compared to elsewhere in the kidney (33). Compared to middle and upper pole stones, the fURS treatment of lower pole stones is more complicated because of the anatomical factors and the limited deflection angle of flexible ureteroscopes (34). Additionally, Tonyali et al. reported that patients with lower pole stones are 2.25 times more likely to have residual stones after fURS compared to patients having stones at other locations (35). Evidently, the spontaneous passing of stone fragments after the surgery was more difficult due to the position of the lower pole. Therefore, we mainly focused on investigating the factors affecting the success rate of the lower calyx calculi.

Nevertheless, in the studies focusing on lower calyceal stones, some researchers have looked at specific factors that can affect the SFR for fURS. Several studies used the Elbahnasy method to calculate the infundibular pelvic angle (IPA) and reported that IPA and other pelvicaliceal anatomy-correlated parameters associate with a lower SFR (36). However, Danuser et al. (37) reported that influence of the collecting system anatomy on disintegrate clearance from the lower calyx could not be demonstrated. The effect of the lower calyx anatomy on the stone-free rate of fURS is still controversial. In addition, controversies also exist on the measurement of pelvicaliceal anatomy. To measure the IPA, urologists need to rely on intravenous urography (IVU) or contrast-enhanced CT (CCT), which are dependent on the use of a contrast agent. First of all, patients with kidney stones generally do not perform these two tests. Moreover, patients with a contrast agent allergy or moderate to severe renal insufficiency are not suitable for these tests. Last but not least, the anatomy of the renal pelvis and calyx will expand with the perfusion of water during the operation, and the pre-operative measurements cannot play an accurate predictive role in the operation. Herein, the IPA was not analyzed in our present study because of the low feasibility in most of the patients.

The current study included several limitations. First, this was a retrospective study, which may cause possible selection bias. Second, the sampling included was relatively inadequate. Moreover, the model was established based on single-center data, and prospective multicenter studies are needed to further validate our results. Finally, due to practical constraints, this paper cannot provide a comprehensive review of multiple stones and lower calyx anatomy, which may be the aim of a future prospective study.

In conclusion, this study indicated that patients with higher radiomics score, smaller stone volume, non-severe hydronephrosis level, and with a more experienced operator were more likely to reach a successful outcome when choosing the flexible ureteroscopy strategy. This clinical–radiomics model may serve as an effective pre-operative prediction method for clinical decision-making for kidney stone patients.

## Data Availability Statement

The original contributions presented in the study are included in the article/Supplementary Material, further inquiries can be directed to the corresponding authors.

## Ethics Statement

The studies involving human participants were reviewed and approved by the Ethics Committee of Tongji Medical College, Huazhong University of Science and Technology. Written informed consent for participation was not required for this study in accordance with the national legislation and the institutional requirements. Written informed consent was not obtained from the individual(s) for the publication of any potentially identifiable images or data included in this article.

## Author Contributions

SW and DH devised the experiment. CL and ZL developed and organized the paper. PL and ZZ designed the tables and figures. QX and HD performed the data analysis. IK and PT participated in the revision of the manuscript. MC and YX wrote the original draft. All authors read and approved the final manuscript.

## Conflict of Interest

The authors declare that the research was conducted in the absence of any commercial or financial relationships that could be construed as a potential conflict of interest.
