# Supplementary material for: A Novel Clinical-Radiomics Model Pre-operatively Predicted the Stone-Free Rate of Flexible Ureteroscopy Strategy in Kidney Stone Patients
Source: Front Med (Lausanne). 2020 Oct 15;7:576925. doi: 10.3389/fmed.2020.576925 (PMC7593485; doi:10.3389/fmed.2020.576925)
Supplement: Supplementary file 1 [file Data_Sheet_1.PDF]

Supplemental materials

Supplement 1: Feature selection methodology

Texture analysis software with algorithms implemented in Matlab 2015a (Math works, Natick, USA) was used for radiomics feature extraction. In our research, a total of 604 radiomics features were extracted from each segmented regions of the CT images and divided into 4 subgroups: Group 1. First order statistics; Group 2. Shape and size-based features; Group 3. Textural features; Group 4. Wavelet features.

Group 1. First order statistics

First-order statistics depict the distribution of voxel intensities using through common and basic metrics within the region of interest (ROI) delineated from CT images.

The following first order features are defined: energy, total energy, entropy, minimum, maximum, 10th percentile, 95th percentile, interquartile range, range, mean absolute deviation (MAD), robust mean absolute deviation (rMAD), mean, standard deviation, variance, median, skewness, kurtosis, root mean square (RMS) and uniformity.

Group 2. Shape and size-based features

In this group of features, we enrolled descriptors of the three-dimensional size and shape of the ROI. These features are independent from the gray level intensity distribution in the ROI and are therefore only calculated on the non-derived image and mask. The following features were calculated: volume, surface area, surface area to volume ratio, sphericity, compactness 1, compactness 2, spherical disproportion, maximum 3D diameter, maximum 2D diameter (Slice), maximum 2D diameter (Column), maximum 2D diameter (Row), elongation, flatness and roundness.

Group 3. Textural features

The texture features mainly represent the pattern and the spatial distribution of the image voxel intensity. A total of 60 kinds of texture features are defined, including 28 features based on Gray Level Co-occurrence Matrix (GLCM), 16 features based on Gray Level Run Length Matrix (GLRLM), 16 features based on Gray Level Size Zone Matrix (GLSZM).

Group 4. Wavelet features

Wavelet feature is to use wavelet transform to decompose the original image. Through the decomposition filter used in X, Y and Z directions of CT image, it may be a low-pass (L) or high-pass (H) filter, arranged in the order of X, Y and Z. There are eight different filtering methods, namely LLL, LLH, LHL, LHH, HLL, HLH, HHL and HHH. Different filtering methods contain first-order statistics, shape features, and statistics-based textural features, so all wavelet features are obtained.

Supplement 2: The R packages used for statistical analysis

The "glmnet" package was used for LASSO binary logistic regression. The "rms" package was used for multivariate binary logistic regression, nomogram construction, calibration curves, and VIF calculation. Calibration curves were constructed using bootstrapping validation with 1,000 resamples. ROC plots and AUC comparison procedures were conducted using the "pROC" package. The Hosmer-Lemeshow test was conducted using the "Resource Selection". Decision curve analysis was performed using the "rmda" package.

Supplement 3: Radiomics score (Rad-score) calculation formula

Rad score=3.598082+age×0.04544301-percent25×0.01118321-SZHGE×0.002397679-LZLGE×2.430904- var×0.004648216- standard deviation×1.721142+LLL Variance1×0.5542491+LLLSZE×0.3824328-LLLLRLGE ×117.3398+LLL median×5.734703E+23-LLHSkewness×0.04958461-LLHSZHGE×0.001678768+LLHSRLGE×19.96994-LHL MaximunProbability×178.6472-LHLKurtosis×0.1092108-LHHMaximunProbability×113.6496-HLLInverseVariance×4.229004-HLHCorrelation×4.072981+HLHGLN1×116.9237+HLHLGRE×41.60477-HLHmean×4267041000000000-HHLSkewness×0.5463918-HHLKurtosis×0.1924146+HHLGLN×143.5697+HHLmean×10890320000000000-HHLmin×0.007632802-HHHContrast1×0.004766524+HHHstandardeviation×1.644575

Supplemental Figure A1: Recruitment pathway for patients in this study.

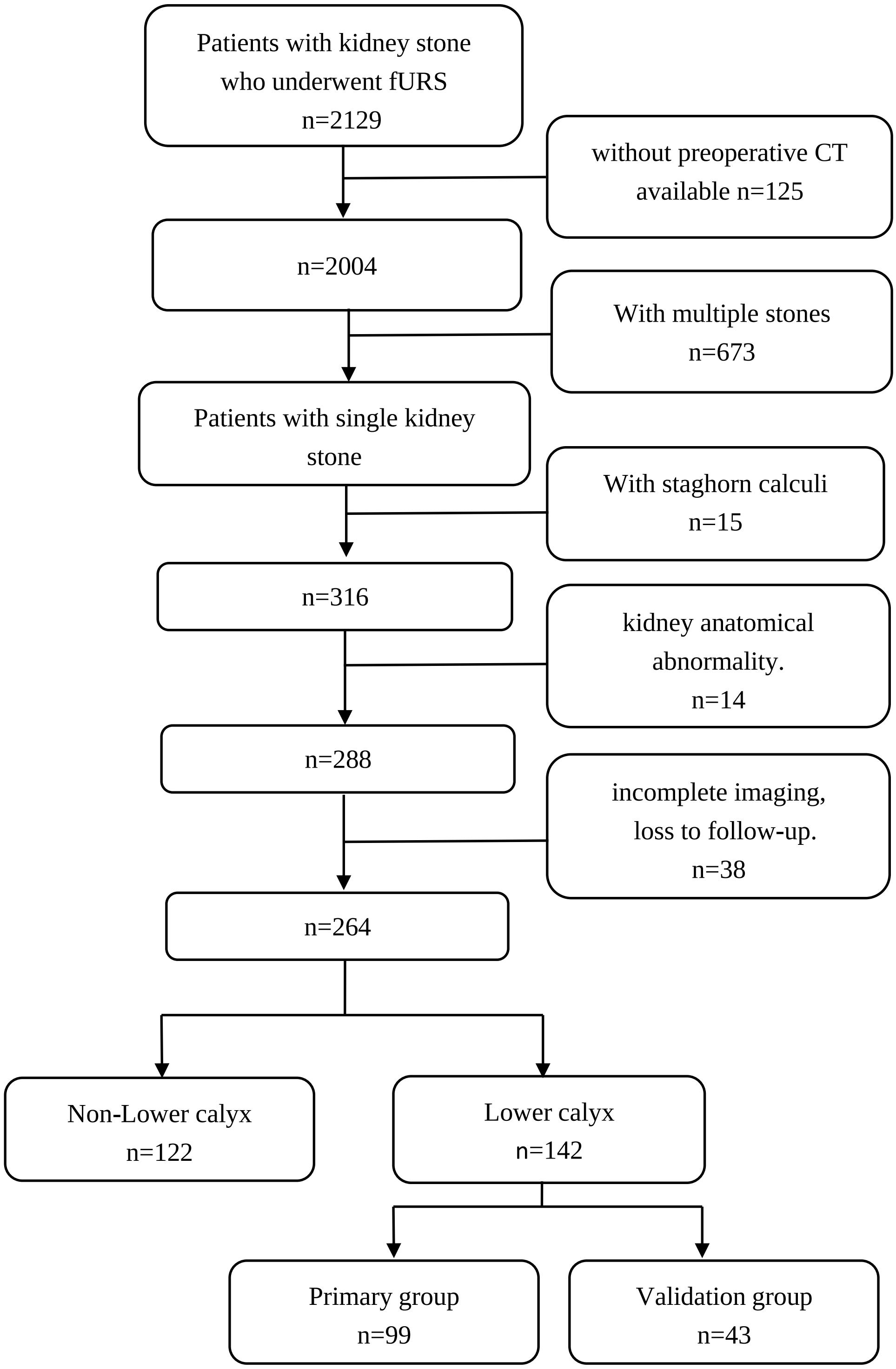

Figure legend

Supplemental Figure A1: Of these 2129 procedures, 1865 were excluded from the study due to the following factors: (a) no non-enhanced MDCT examination before fURS; (b) presence of multiple stones or staghorn calculi; (c) kidney anatomical abnormality: transplant kidney, solitary kidney, horseshoe kidney and duplication; (d) incomplete imaging, and lost to follow-up. Staghorn calculi were defined as the large size of the renal pelvis calculi that protrudes into at least one renal calyx in the present study.

Supplemental Figure A2: Distributions of the radiomics score and postoperative outcome for each patient in the primary and validation groups.

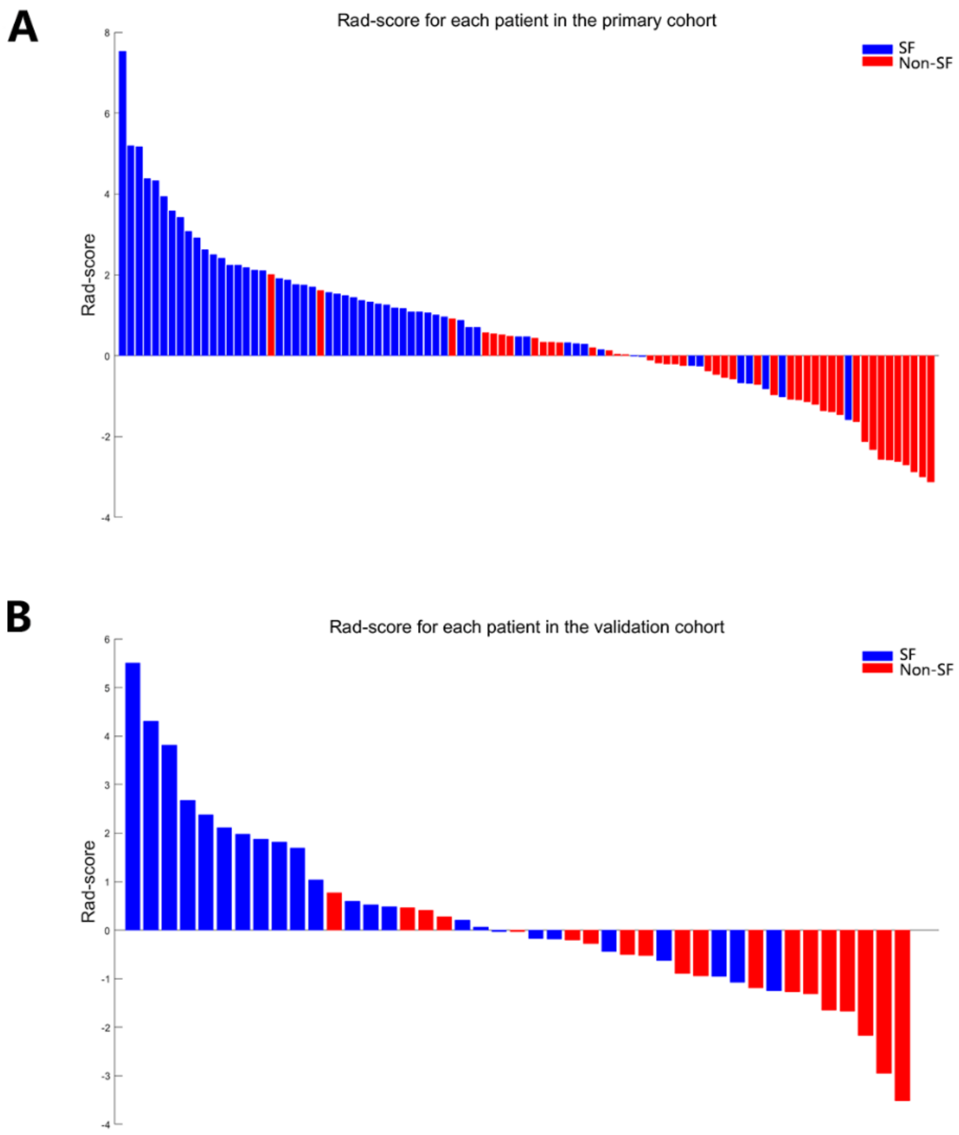

Supplemental Figure A2: A. Rad-score for each patient in the primary cohort; B. Rad-score for each patient in the validation cohort. The postoperative outcome is marked with different colors. The blue columns depict the stone-free (SF) cases, and those red depict non-stone-free (Non-SF) cases.
